# Constructing Moral Selves Through Food: A Qualitative Study of Orthorexic Eating Practices in the UK

**DOI:** 10.3390/bs16060997

**Published:** 2026-06-15

**Authors:** Panagiota Tragantzopoulou, Elina Mitrofanova, Vaitsa Giannouli

**Affiliations:** 1 School of Social Sciences, University of Westminster, London W1B 2HW, UK; 2 School of Human Sciences, University of Greenwich, London SE10 9LS, UK; e.mitrofanova@greenwich.ac.uk; 3 School of Psychology, Aristotle University of Thessaloniki, 541 24 Thessaloniki, Greece; giannouliv@hotmail.com

**Keywords:** orthorexia nervosa, moralization of food, healthy eating, dietary identity, qualitative research, healthism, eating behaviours

## Abstract

Contemporary health cultures increasingly promote disciplined eating and bodily optimisation, contributing to growing interest in orthorexia nervosa (ON), a pattern of restrictive eating characterised by an obsessive focus on food purity and health. While ON has been widely studied in relation to dietary restriction and health anxiety, less attention has been given to how individuals themselves construct meaning around these practices. The present qualitative study aimed to explore how individuals displaying orthorexic tendencies construct moral identity and self-worth through their dietary practices. Eighteen participants (13 women, 5 men; aged 19–58) living in the United Kingdom who self-identified as “healthy eaters” took part in semi-structured interviews. Data were analysed using reflexive thematic analysis within a social constructionist framework. Four themes were generated: (1) The Disciplined Self, describing how strict dietary practices were framed as evidence of personal control and self-regulation; (2) The Body as Evidence of Purity and Health, where physical appearance and bodily feelings were interpreted as confirmation of moral and dietary correctness; (3) Ethical Eating and Moral Positioning, illustrating how participants positioned their food choices as ethically superior; and (4) Guilt and Moral Repositioning, highlighting the moral emotions that followed perceived dietary transgressions. These findings suggest that orthorexic eating practices function not only as health behaviours but also as moral performances through which individuals construct disciplined, responsible, and virtuous identities. Understanding these moral and identity dimensions may help situate orthorexic tendencies within broader sociocultural narratives surrounding health, morality, and self-discipline.

## 1. Introduction

Across historical periods, dominant ideals regarding body shape and dietary practices have shifted considerably, reflecting broader cultural, social, and economic transformations. These shifts have contributed to changing standards of physical appearance and health behaviours, often promoting new forms of bodily regulation and self-surveillance (Tragantzopoulou, 2021). In contemporary societies, increasing emphasis is placed on self-improvement, personal responsibility for health, and the pursuit of bodily optimisation. Within this sociocultural context, body dissatisfaction has risen in recent decades, partly driven by the proliferation of appearance standards that emphasise a lean, muscular, and “fit” body as an ideal for both men and women (Louw et al., 2025; Adamidou & Tragantzopoulou, 2025).

Against this backdrop, orthorexia nervosa (ON) has emerged as a phenomenon attracting growing academic and clinical attention. ON is generally described as an obsessive focus on consuming foods perceived as healthy, pure, or clean, accompanied by rigid dietary rules and feelings of anxiety, distress, or guilt when these rules are violated (Cheshire et al., 2020; Zickgraf, 2020). Unlike other eating disorders (EDs) that primarily focus on weight loss or body size, ON is characterised by a fixation on food quality and purity. In relation to anorexia nervosa (AN), orthorexia nervosa shares several psychological features such as perfectionism, cognitive rigidity, and a strong need for control. However, it differs fundamentally in its focus: whereas AN is primarily characterised by an intense fear of weight gain, body image disturbance, and restriction of caloric intake, ON is centred on the perceived purity and quality of food rather than its quantity or energy content (Dunn & Bratman, 2016; Koven & Abry, 2015). However, the distinction between health-conscious behaviour and pathological restriction remains contested, particularly within contemporary societies where public discourse around “correct” or “optimal” eating practices is highly visible. Orthorexic tendencies have also been observed in contexts where body control and performance optimisation are central, such as athletic populations, where strict dietary regulation may be used to enhance perceived physical performance and bodily efficiency (Uriegas et al., 2021).

Despite growing research interest, ON is not currently recognised as a formal diagnostic category in either the Diagnostic and Statistical Manual of Mental Disorders or the International Classification of Diseases. Nevertheless, it has been widely investigated, with scholars attempting to clarify its diagnostic features, risk factors, and underlying motivations (Dunn & Bratman, 2016; Donini et al., 2022, Tragantzopoulou & Giannouli, 2024). Existing research suggests that the motivations underpinning restrictive “clean eating” practices are complex and cannot be fully explained by simplified diagnostic criteria (Cinquegrani & Brown, 2018; Greville-Harris et al., 2020; Fixsen et al., 2020; McGovern et al., 2020; Valente et al., 2020b). Research also suggests that orthorexic dietary practices may serve important psychological and social functions. For instance, Mitrofanova et al. (2021b) found that adherence to self-defined healthy diets was frequently associated with desires for control, identity formation, and the management of life transitions. In this context, dietary regulation may provide individuals with a sense of agency and coherence during periods of uncertainty or personal change.

One area that has received particular attention in the ON literature concerns the relationship between orthorexic tendencies and plant-based diets. Veganism and other plant-based dietary practices have grown substantially in popularity in recent decades, driven by a range of motivations including environmental concerns, animal welfare activism, and perceived health benefits (Kalika et al., 2022; Szulc et al., 2025). This shift has also been influenced by social media and evolving dietary trends that encourage individuals to closely monitor and regulate their food intake (Szulc et al., 2025). Within this context, researchers have begun to examine whether plant-based diets are associated with orthorexic behaviours.

Empirical findings in this area remain mixed. Some studies report higher orthorexic tendencies among individuals following vegetarian or vegan diets (Brytek-Matera, 2021). However, other research suggests that motivations for plant-based eating play a crucial role in shaping this relationship. For example, Barthels et al. (2018) found that veganism motivated primarily by ethical concerns such as animal welfare may act as a protective factor against pathological eating behaviours, whereas motivations centred on health optimisation, aesthetics, or bodily healing were more strongly associated with orthorexic tendencies. These findings highlight the complexity of interpreting restrictive dietary practices, as plant-based diets may be adopted for diverse ethical, environmental, or health-related reasons. Consequently, some scholars caution that framing such dietary patterns primarily through a pathological lens risks oversimplifying the social and moral meanings attached to them and may contribute to the misinterpretation or medicalization of alternative dietary lifestyles (Fixsen et al., 2025a).

Qualitative research has further contributed to understanding the lived experiences underlying orthorexic practices. For example, Fixsen et al. (2020) describe how the pursuit of an idealised body may motivate individuals to adopt restrictive dietary practices that gradually become central to everyday life. Similarly, McGovern et al. (2020), in interviews with individuals who identified as recovered from ON, found that external influences—including family environments, healthcare practitioners, and alternative health communities—often played a significant role in the development of orthorexic behaviours. These studies suggest that ON frequently intersects with broader social environments and cultural expectations surrounding health and self-discipline.

From a sociocultural perspective, orthorexic practices can also be interpreted through broader cultural discourses surrounding health and responsibility. One influential concept in this regard is healthism, originally described by Crawford (1980), which refers to an ideology in which health becomes a central moral value and a primary measure of personal worth. Within this framework, individuals are expected to actively manage their health through lifestyle choices, thereby transforming health into a form of personal responsibility and moral obligation. Scholars have argued that these discourses are closely aligned with broader processes of neoliberal individualism, where the pursuit of health functions both as a marker of self-discipline and a form of moral citizenship (Kristensen et al., 2016; Hehlmann et al., 2018).

Building on this perspective, qualitative research has explored ON through the lens of bio-citizenship, examining how individuals construct identities around responsible health behaviours and dietary practices. For example, work by Fixsen and colleagues highlights how strict healthy eating practices can function as markers of moral responsibility, professional competence, and self-discipline within environments saturated with health messaging and food marketing (Fixsen et al., 2025b). In this context, dietary practices become ways through which individuals perform responsible citizenship by demonstrating commitment to bodily optimisation and self-regulation. However, this work also reveals an important paradox: behaviours that are socially encouraged as responsible health practices may simultaneously be interpreted as pathological when they become excessively rigid or restrictive.

Recent sociological scholarship has further emphasised how such dynamics are embedded within contemporary wellness cultures. Within societies increasingly shaped by neoliberal ideals of self-optimisation and personal responsibility, health practices often function as forms of moral governance in which individuals are expected to regulate their bodies and lifestyles in pursuit of idealised standards of wellbeing (Horovitz, 2025). In this framework, practices such as “clean eating” operate not only as dietary choices but also as symbolic expressions of discipline, purity, and self-mastery. However, while this perspective provides valuable insight into the broader ideological and structural forces shaping contemporary health behaviours, it primarily conceptualises orthorexia at the level of cultural discourse and moral governance. Less attention has been given to how individuals themselves interpret, negotiate, and embody these moral expectations in their everyday dietary practices.

Closely related to these processes is the moralization of food practices. Moralization refers to the process through which personal preferences gradually acquire moral significance, transforming individual choices into matters of right and wrong (Rozin, 1999). Within contemporary wellness cultures, foods are frequently categorised as “clean,” “pure,” or “toxic,” reinforcing binary distinctions between morally acceptable and unacceptable consumption practices. Recent neurocognitive research supports this perspective, demonstrating that individuals tend to implicitly associate foods with moral attributes, linking “healthy” foods with virtue and high-calorie foods with moral negativity (Lakritz et al., 2022). These findings suggest that dietary choices may become deeply embedded in moral evaluation processes.

Food practices also play a central role in identity construction. As Fischler (1988) argues, food occupies a key position in both collective and individual identity, shaping how individuals define themselves and distinguish themselves from others. Through dietary practices, individuals symbolically communicate values, ethical commitments, and lifestyle orientations. Consequently, practices such as veganism, clean eating, or strict nutritional regimes may function not only as health behaviours but also as expressions of moral identity.

Recent qualitative syntheses further reinforce the importance of these sociocultural dynamics. For example, Tragantzopoulou et al. (2026a), in a qualitative meta-synthesis of ON research, conclude that orthorexic behaviours are deeply intertwined with broader cultural ideals surrounding health, control, and moral worth. Rather than being experienced purely as pathological, these behaviours may simultaneously function as coping strategies, identity performances, and socially reinforced practices. Such findings suggest that orthorexia cannot be fully understood solely through the lens of individual psychopathology. Instead, it appears to emerge at the intersection of personal motivations, cultural ideals of health and appearance, and broader sociopolitical discourses surrounding responsibility for bodily self-care.

From a historical and psychological perspective, restrictive eating behaviours have also been interpreted through traditions of asceticism and extreme self-denial. Classical analyses of AN situate such behaviours within broader religious and ascetic traditions, where fasting and bodily control were historically linked to spiritual purity and moral elevation, sometimes extending to life-threatening self-starvation, as illustrated in accounts of saintly figures such as Catherine of Siena (Rampling, 1985; Vitousek et al., 1998). This highlights that extreme dietary restraint has long been embedded in cultural and moral frameworks rather than being a purely modern clinical phenomenon.

Despite growing scholarly attention to ON, relatively little qualitative research has examined how individuals themselves interpret and narrate their dietary practices, particularly in relation to moral identity and self-evaluation. While previous studies have explored motivations for healthy eating, the social influences surrounding orthorexic behaviours or the nutritional impact (Fixsen et al., 2020, Cheshire et al., 2020; Valente et al., 2020a; Mitrofanova et al., 2021a; Mitrofanova et al., 2021b), fewer have investigated how individuals position themselves as morally responsible subjects through their everyday food practices. Understanding these processes is important for clarifying how health-oriented dietary practices become imbued with moral meaning and personal significance.

Therefore, the present study explores how individuals displaying orthorexic tendencies construct and negotiate moral meanings around their dietary practices. Specifically, it examines how food choices become sites of moral self-construction, discipline, and embodied virtue. The study addresses the following research question: How do individuals with orthorexic tendencies construct themselves as morally worthy subjects through their dietary practices? By focusing on the moral and identity-related dimensions of eating practices, this analysis seeks to contribute to ongoing debates regarding the cultural meaning of orthorexia and the broader social contexts in which such behaviours emerge.

## 2. Materials and Methods

### 2.1. Study Design

The present study employed a qualitative research design to explore how individuals who strongly identify with healthy eating construct meaning around their dietary practices. Qualitative methodologies are particularly suited to examining complex experiences, beliefs, and motivations that cannot be fully captured through quantitative approaches. By allowing participants to describe their perspectives in their own words, qualitative research provides rich insights into the social and psychological meanings attached to behaviours and practices (Lim, 2025).

The study was informed by a social constructionist epistemological perspective. Social constructionism assumes that knowledge and meaning are not fixed or objectively determined but are produced through social interaction, cultural norms, and shared discourses (Burr & Dick, 2025). Within this framework, eating practices and beliefs about “healthy” food are understood as socially and culturally situated rather than purely individual or biomedical phenomena. This perspective is particularly suited to examining the moral and identity-related dimensions of eating, as notions of “healthy” and “unhealthy” are often imbued with moral value and linked to self-discipline, responsibility, and personal worth (Fixsen et al., 2025b). Such meanings are shaped through broader societal discourses surrounding health, nutrition, and lifestyle, which influence how individuals construct and regulate their eating behaviours (Tragantzopoulou et al., 2024a). Adopting this approach enabled the study to explore how participants negotiate meanings around healthy eating, discipline, morality, and identity within their social contexts.

### 2.2. Participants and Recruitment

A total of 18 participants took part in the study (13 women and 5 men), ranging in age from 19 to 58 years (see Table 1). No participants withdrew after recruitment, and no individuals who met inclusion criteria declined participation. The sample size was considered appropriate in relation to the study aims and analytic approach. The study did not seek to achieve data saturation. As argued by Braun and Clarke (2019), the concept of saturation assumes that meaning can be fully captured and exhausted, which is not consistent with reflexive approaches that view analysis as an interpretative and ongoing process. Rather than aiming for completeness, the focus was on developing rich and conceptually meaningful interpretations of participants’ accounts. The adequacy of the sample was therefore guided by the concept of “information power” (Malterud et al., 2016), whereby the more relevant and information-rich the sample is in relation to the research question, the fewer participants are required. In the present study, the specificity of the sample, the focused research aims, and the depth of the interview data contributed to a dataset with sufficient analytic richness to support the development of robust themes.

All participants were living in the United Kingdom (UK) at the time of the study. Recruitment of individuals exhibiting orthorexia-related tendencies presents methodological challenges due to the absence of formally recognised diagnostic criteria or validated diagnostic tools. In the present study, participants were therefore recruited based on self-identification as individuals who strongly adhere to healthy eating practices. To enhance consistency in inclusion, the research team applied a set of informal screening criteria during initial contact. Specifically, participants were asked to briefly describe their typical daily diet, their motivations for following a “healthy” diet, and the degree of flexibility in their eating patterns. Individuals were included if their responses reflected: (a) strong adherence to self-imposed dietary rules, (b) rigid or consistent food choices with limited variation, and (c) a clear cognitive or emotional investment in maintaining these practices (e.g., feelings of satisfaction, control, or distress linked to adherence). These criteria were discussed among the research team to ensure a shared understanding of eligibility and to promote consistency across recruitment decisions. In addition, the research team considered participants’ descriptions of their eating behaviours and attitudes during initial contact. Participants typically reported adherence to a self-defined “healthy” diet, expressed strong and specific beliefs about the health properties of particular foods, and described relatively little variation in their daily dietary practices. Many participants also indicated that their sense of satisfaction or wellbeing was closely connected to their ability to adhere to their chosen dietary rules.

A purposeful snowball sampling strategy was used to recruit participants. Participants were approached through online advertisements (e.g., social media platforms) and via email or direct messaging following expressions of interest. Initial participants were identified through the researchers’ professional and social networks, as well as individuals who had previously expressed interest in topics related to nutrition and wellbeing. Efforts were made to approach individuals with varying dietary approaches (e.g., plant-based diets, “clean eating,” fitness-oriented diets) in order to capture a range of experiences and motivations associated with healthy eating. These individuals were then asked to share information about the study with others who might be interested in participating. To mitigate potential homogeneity associated with snowball sampling, recruitment was supplemented with online advertisements posted on relevant platforms (e.g., social media groups focused on health and nutrition). This approach aimed to broaden the sample beyond immediate networks and include participants with diverse backgrounds and perspectives. Participants received an information sheet outlining the aims and procedures of the study. Due to the lack of formal diagnostic criteria for orthorexia nervosa, the information sheet used neutral language and did not explicitly refer to ON in order to avoid influencing participants’ responses.

No restrictions were placed on ethnicity, occupation, or socio-demographic background. Exclusion criteria included current or previous ED diagnoses, other psychiatric diagnoses, inability to speak English, and age under 18 years. Exclusion criteria were assessed through self-report during initial contact and confirmed at the beginning of the interview, where participants were asked whether they had ever received a formal diagnosis of an ED or other psychiatric condition. Individuals with current or past ED diagnoses were excluded in order to reduce conceptual and clinical overlap between orthorexia-related tendencies and clinically recognised eating disorders. This decision was made to allow a focused exploration of restrictive healthy eating practices as a distinct phenomenon outside established clinical categories. However, we acknowledge that this approach may have excluded individuals at the more severe or clinically significant end of the spectrum, where orthorexic behaviours may overlap with or co-occur alongside EDs. As such, the findings should be interpreted as reflecting subclinical or non-diagnosed experiences rather than the full continuum of orthorexia-related presentations.

### 2.3. Data Collection

Data collection took place between June 2021 and March 2023, during the post-COVID-19 period. This temporal context is important, as ongoing public health discourse around immunity and wellbeing may have influenced participants’ understandings of healthy eating and lifestyle practices. Data were collected through semi-structured interviews using open-ended questions designed to explore participants’ experiences, motivations, and beliefs surrounding healthy eating. Eight interviews were conducted face-to-face in locations chosen by the participants. The remaining interviews were conducted online, enabling individuals living in different areas of the UK to participate and increasing the accessibility of the study. Only the participant and the researcher were present during the interviews.

Interviews were conducted by two members of the research team. The first author (female, PhD) conducted the online interviews, while the second author (female, PhD) conducted the face-to-face interviews. Both researchers are academics with experience in qualitative and mixed-methods research and have received formal training in qualitative interviewing techniques. No prior relationship was established with participants before the study commenced. During recruitment and at the beginning of each interview, participants were informed that the researchers were interested in exploring healthy eating practices and individuals’ experiences in relation to these. The researchers’ interest in the topic was grounded in their academic engagement with eating behaviours and qualitative research.

At the beginning of each interview, participants were informed about the purpose of the study and were reminded that their participation was voluntary. A brief statement was read aloud assuring participants of confidentiality, encouraging them to speak openly without fear of judgement, and emphasising their right to withdraw from the interview at any time without consequence.

Interviews followed a semi-structured interview guide informed by existing literature on ON and healthy eating practices. Questions were designed to encourage participants to reflect on their relationship with food, including topics such as: (a) what healthy eating means to them, (b) how their dietary practices developed, (c) the motivations underlying their food choices, (d) their beliefs about the health effects of specific foods, (e) and the emotional experiences associated with adhering to or deviating from their dietary practices. The semi-structured format allowed participants to elaborate on issues that were personally meaningful while ensuring that key topics were explored across interviews.

All interviews were audio-recorded with participants’ consent and lasted approximately one hour on average. Field notes were taken during and immediately after each interview to capture contextual observations and initial reflections. At the end of each interview, participants were debriefed (a debrief sheet, which included information on relevant mental health support services available in the UK, was provided), and any questions about the study were addressed. No repeat interviews were conducted. Transcripts were returned to participants for optional comment or correction. Two participants responded with minor clarifications and additional examples related to their experiences.

### 2.4. Data Analysis

Interview data were analysed using reflexive thematic analysis, following the approach outlined by Braun and Clarke (2019). Thematic analysis is a flexible qualitative method that enables the identification, analysis, and interpretation of patterns of meaning across a dataset. The analysis was primarily inductive, in that themes were developed through close engagement with the data. However, consistent with Braun and Clarke’s (2019) reflexive thematic analysis approach, the analysis was also theoretically sensitised, meaning that the researchers’ prior engagement with relevant literature (e.g., healthism, moralization, and sociocultural perspectives on eating) inevitably informed interpretation, while care was taken to ensure that themes remained grounded in participants’ accounts.

The first author (PT) began by reading and re-reading the interview transcripts in order to become familiar with the full dataset. Initial open coding was then conducted to identify meaningful segments of text representing participants’ experiences, beliefs, and interpretations related to healthy eating practices. Codes with conceptual or semantic similarities were subsequently grouped together into preliminary sub-themes. Through an iterative process of comparison and refinement, these sub-themes were further developed and combined into broader themes that captured key patterns within the data. Coding was primarily conducted by the first author, with ongoing analytic input from the second (EM) and third (VG) authors, who reviewed codes and contributed to the development and refinement of themes. Throughout the analysis process, the emerging coding framework and thematic structure were reviewed and discussed with the second and third authors. This collaborative process helped ensure that the themes accurately reflected the dataset and enhanced the credibility of the analysis. A coding tree was developed during the analytic process, illustrating the progression from initial codes to sub-themes and overarching themes. Data analysis was conducted manually without the use of qualitative data analysis software, consistent with the reflexive and interpretative nature of the analytic approach. Participants did not provide feedback on the final themes or findings; however, transcripts were returned for optional comment or clarification, as described above.

The study adhered to the Consolidated Criteria for Reporting Qualitative Research (COREQ) guidelines (Tong et al., 2007) in order to ensure transparency and rigour in the reporting of qualitative methods (see Appendix A).

### 2.5. Researcher Reflexivity

In qualitative research, the researcher plays an active role in the interpretation of data (Lim, 2025); therefore, reflecting on the researcher’s positionality is an important component of methodological transparency. The research team approached the study from a perspective that recognised eating practices as socially and culturally situated behaviours, rather than viewing them solely through a clinical or biomedical framework. The authors come from backgrounds in psychology and health research, with prior academic and research engagement in eating behaviours, body image, and the broader field of eating disorders. As such, they were familiar with both clinical and sociocultural debates surrounding ON and healthy eating practices. While none of the researchers identified as having lived experience of ON, they acknowledged that their academic training and exposure to public health and wellness discourses may incline them to view dietary regulation through lenses of risk, discipline, and moralization. In addition, the researchers recognised that their own relationships with food and culturally normative healthy eating practices were not the focus of the study. could shape assumptions about what constitutes balance, control, or excess. This awareness informed a reflexive stance throughout the study, particularly in actively resisting the pathologization of participants’ behaviours and instead focusing on how meanings around health, morality, and identity were constructed within participants’ accounts. Throughout the research process, particular attention was given to maintaining reflexivity, meaning that the researchers remained aware of how their own perspectives, assumptions, and disciplinary backgrounds might shape the interpretation of participants’ accounts. The use of open-ended interview questions, inductive coding procedures, and collaborative discussions among the research team helped ensure that themes were grounded in participants’ narratives rather than imposed by pre-existing theoretical expectations. Regular discussions between members of the research team during the analytic process also helped to challenge individual interpretations, refine coding decisions, and ensure that the final thematic structure reflected the breadth and complexity of the data.

### 2.6. Ethical Considerations

Ethical approval for this study was obtained from the relevant University Research Ethics Committee prior to data collection. All procedures were conducted in accordance with established ethical guidelines for research involving human participants. Participants received a detailed information sheet explaining the purpose of the study, what participation would involve, and how their data would be used. Written informed consent was obtained from all participants before the interviews commenced. Participants were informed that their participation was voluntary and that they had the right to withdraw from the study at any stage without providing a reason.

To protect confidentiality, all identifying information was removed from interview transcripts and pseudonyms were assigned to participants. Audio recordings and transcripts were stored securely and were accessible only to members of the research team. Given that discussions surrounding food, health behaviours, and personal experiences may occasionally evoke emotional responses, participants were reminded that they could pause or terminate the interview at any time. At the end of each interview, participants were debriefed, and any questions or concerns regarding the study were addressed.

## 3. Results

The analysis identified recurring discursive patterns through which participants framed dietary practices in terms of discipline, purity, responsibility, and self-control (see Table 2). Participant quotations are presented throughout the findings to illustrate and support each theme, with each quotation identified by participants’ pseudonyms. The findings are grounded in the data and reflect consistent patterns across participants’ accounts. Major themes are presented clearly, alongside attention to variations and nuanced experiences within the dataset where relevant.

### 3.1. The Disciplined Self

Across participants’ accounts, healthy eating practices were frequently framed as less a matter of nutritional knowledge and more a matter of mental discipline and self-regulation. Participants repeatedly positioned the mind as the central mechanism through which dietary practices could be maintained, suggesting that successful adherence to restrictive or demanding eating practices depended primarily on psychological strength and determination. Several participants described dietary restriction and fasting as tests of personal resilience, emphasising the capacity to push oneself beyond perceived limits. Daniel described how engaging in these practices functioned as a form of self-challenge:

*“It’s always like a test… it helps me push myself even more… if you’re mentally strong and can sort of push yourself then for me it helps in all other aspects of my life.”*

In this account, dietary discipline is constructed not simply as a health practice but as a broader exercise in character development, where the ability to tolerate physical discomfort or deprivation becomes evidence of mental strength. Food practices therefore extend beyond bodily regulation and become symbolic of a wider capacity for perseverance, self-improvement, and control across different areas of life.

Participants often emphasised the primacy of the mind over the body, suggesting that physical hunger or difficulty could be overcome through psychological adjustment. For example, fasting was described as something that initially “shocks” the body but ultimately becomes manageable once the mind adapts:

*“To be honest, for me, it’s all in the mind, because friends of mine always used to say “ ah! “. Like they’ll even forget that I’m fasting and they’ll be eating and they’ll be like: “oh no sorry! But I didn’t realize…” but for me I’ll be like: “nah, don’t worry about it” cause it’s in the mind. … once your body is adapted and your mind is adapted to it… it becomes less difficult… it’s not easy though, it is testing.”*

(Daniel)

Here, bodily needs are framed as secondary to mental determination, reinforcing the idea that the body can be trained or disciplined through willpower. This framing positions dietary restriction as a form of self-mastery in which physical sensations are subordinated to cognitive control.

The theme of mental strength as a prerequisite for health was echoed across multiple accounts. Participants frequently suggested that maintaining strict dietary practices required a particular type of mindset or level of commitment. As Maya succinctly stated:

*“Body can adjust if they have strong will but you have to probably put your effort in it.”*

Similarly, Isabella emphasised that the ability to maintain dietary discipline ultimately depended on being “*disciplined enough.”* These statements construct healthy eating not merely as a behavioural choice but as an indicator of personal virtue and determination, implying that success in maintaining dietary regimes reflects internal qualities such as willpower and resilience.

Participants also described perceived psychological benefits of dietary restriction or “clean eating,” often linking these practices to improved cognition and emotional clarity. Lucas explained that eating in this way produced a sense of heightened mental functioning:

*“I have better mental clarity… I can think clearer, it’s like a cloud from your mind that has vanished just because you are eating cleaner.”*

Such accounts further reinforce the perceived relationship between dietary control and psychological optimisation, where food practices are understood as directly shaping mental states and cognitive performance.

In some accounts, this connection between food and mindset extended to broader lifestyle changes. Participants suggested that adopting restrictive or health-focused diets could initiate a wider transformation in attitudes and behaviours:

*“What you eat… you change your mindset a little bit… you start thinking about workout and drinking less and just choosing healthy.”*

(Laura)

Within this framing, dietary practices become part of a holistic self-regulation project, in which individuals cultivate discipline across multiple domains of life, including exercise, alcohol consumption, and general lifestyle choices.

### 3.2. The Body as Evidence of Purity and Health

Participants frequently described dietary practices as producing visible and embodied transformations, positioning the body as a site through which the benefits of “*healthy*” eating could be observed, measured, and validated. Improvements in physical appearance, bodily sensations, and perceived vitality were often interpreted as confirmation that particular dietary choices were correct or beneficial. For some participants, dietary practices were closely tied to managing the body’s appearance and performance in everyday life. Natalie, who worked as a model, explained that eating lightly was necessary to maintain a desired physical presentation:

*“Especially if you are doing like an underwear shoot… if I have a big breakfast sometimes I get bloated… so I like to keep it light in the morning.”*

Here, food consumption becomes something that must be carefully regulated to ensure the body remains visually acceptable and functional within particular social and professional contexts. Similarly, others described how dietary changes were associated with improvements in bodily appearance and self-perception. Amira reflected that through healthier eating, they felt more positively toward their body:

*“I look my best and I admire my body for doing so much for me and I feel good about myself.”*

Across several accounts, participants described noticeable changes in skin, energy levels, and overall physical condition, interpreting these shifts as direct outcomes of dietary behaviour. For example, Sofia reflected on a past period of consuming large amounts of sugar and processed foods, describing how this had negatively affected their energy and appearance:

*“I get really tired easy… slowly it’s changed to salads… it’s good for your skin and everything.”*

Similarly, Maya linked dietary adjustments to improvements in skin health and other bodily symptoms:

*“I had really bad skin… so much acne… and that all disappeared once I stopped dairy and honey.”*

In these narratives, dietary change is understood as producing visible bodily improvements that function as evidence of health and self-care. Weight loss was also framed as meaningful even when the objective change was relatively small. Aisha explained:

*“I am really happy because with healthy eating I have lost some weight… it’s not that much weight that would make a difference, but for me it does, it boosts my confidence.”*

Within such accounts, bodily transformation becomes intertwined with self-esteem and identity, reinforcing the idea that dietary practices shape not only physical health but also psychological wellbeing and self-worth.

Participants also described the body as a site through which internal purity or contamination could be perceived. One striking way this was expressed was through discussions of bodily smell, where dietary choices were believed to produce detectable sensory differences between individuals. Maya suggested that they could identify people’s diets through their scent:

*“I can see when people are really heavy meat eaters… I can sense it from them… they smell, they really do and they don’t know.”*

This framing constructs dietary practices as something that not only shapes internal bodily processes but also produces outward sensory markers that distinguish individuals from one another. Food choices therefore become part of a broader moral and bodily boundary-making process, where some bodies are implicitly framed as cleaner or more desirable than others.

A similar concern with bodily purity emerged among participants who described avoiding certain foods, such as garlic or onions, due to their perceived effects on the body’s internal balance and external smell. Natalie explained:

*“I want to keep my body as natural as possible… if I kill some bacteria that is bad I also kill bacteria that’s good.”*

Here, dietary restriction is framed as a way of preserving the body’s natural state and protecting it from substances perceived as disruptive or contaminating. In this way, food practices become linked to broader ideals of bodily purification and natural balance.

However, while participants often emphasised the benefits of dietary restriction, some accounts also revealed moments of ambivalence. For example, Maya acknowledged that although they felt “cleaner” after dietary changes, they also experienced reduced physical strength:

*“I feel cleaner, but I feel weaker too… I used to run much more and I can’t run now.”*

Similarly, Isabella described significant fatigue when adopting a restrictive diet:

*“For the first week I was very weak and cold all the time… there is a lack of energy because you do not have anything animal-related in your body.”*

These accounts suggest that while dietary practices were often associated with ideals of bodily improvement and purity, participants also encountered tensions between perceived health benefits and physical limitations. Nevertheless, these difficulties were frequently interpreted as temporary adjustments or issues requiring further dietary optimisation rather than reasons to question the underlying value of the dietary regime.

### 3.3. Ethical Eating and Moral Positioning

For many participants, dietary practices were not framed solely in terms of health or bodily outcomes but were also embedded within broader ethical and moral considerations. In particular, vegan and vegetarian participants described their food choices as connected to concerns about animal welfare, exploitation, and the moral implications of food production. These narratives positioned dietary behaviour as an expression of personal values and ethical responsibility. Several participants described moments of moral awakening that prompted them to reconsider their relationship with animal products. Exposure to information about animal treatment was frequently cited as a turning point. Laura reflected on how witnessing representations of slaughterhouse practices reshaped their understanding of animals:

*“When you do see how animals are being treated and being killed in a slaughterhouse… you start changing the way you thought. Animals are actually equal to us… it’s not a subject that should obey us, that should serve us.”*

Similarly, Zoe described how watching online material about the meat industry triggered feelings of disgust and prompted them to reconsider their dietary habits:

*“It all started when I watched a YouTube video about how animals are treated… I got disgusted with the way the meat industry operates just for money.”*

In these accounts, dietary change is framed as a response to ethical awareness, where participants reinterpret everyday food practices through a moral lens. Avoiding animal products therefore becomes a way of aligning behaviour with newly recognised ethical principles. Interestingly, several participants also emphasised that their current dietary positions contrasted with earlier eating habits. The majority of the vegans and vegetarians had previously enjoyed eating meat:

*“… I used to love meat and get excited every time my mum would cook it.”*

(Ethan)

This retrospective contrast highlights how dietary identity can shift over time as individuals reinterpret past behaviours through new moral frameworks. At the same time, participants appeared aware that ethical dietary choices can generate social tensions. Some described how strongly expressed vegan advocacy could provoke negative reactions from others. Laura reflected on the risk of creating antagonism through overly critical messaging:

*“Otherwise we would just create lots of ‘vegan hatred’… some people are so extreme, criticise all the time… other meat-eating people would hate vegans.”*

This comment suggests an awareness that moral positioning around food can produce social divisions, particularly when dietary practices become associated with judgement or perceived moral superiority. In response, some participants emphasised a more flexible and non-prescriptive stance toward others’ eating behaviours. For example, Natalie explained:

*“I know what I want for me, but I would never tell other people how to behave… people have to make their own experience.”*

Here, dietary choices are framed as a personal ethical commitment rather than something that should be imposed on others. This positioning allows participants to maintain their moral stance while also avoiding conflict or accusations of moralising.

Participants also reflected on the complexity of ethical consumption beyond food. Maya questioned the selective moral outrage sometimes expressed around animal rights, highlighting inconsistencies in broader patterns of consumption:

*“Why do you protest animal testing when you go and buy from Primark?… you pay companies that exploit people working in horrible conditions… that is also a form of slavery.”*

Through this critique, the participant expands the ethical conversation beyond dietary choices to include issues such as labour exploitation and global inequality. This reflection suggests that participants were not only negotiating the morality of food consumption but were also grappling with broader questions about ethical consistency in everyday consumer practices.

### 3.4. Guilt and Moral Repositioning

While participants frequently described their dietary practices in terms of discipline, bodily optimisation, and ethical commitment, moments of deviation from established rules revealed the moral intensity attached to these practices. When participants departed from their dietary routines—whether by consuming “unhealthy” foods, eating animal products, or missing exercise—they often described feelings of guilt, discomfort, and a perceived loss of control. These experiences illustrate how food practices were embedded within a broader moral framework, where adherence symbolised self-discipline and virtue, and deviation triggered a need for correction. For some participants, deviations were experienced not only psychologically but also physically. Consuming foods perceived as unhealthy was described as producing immediate bodily discomfort, reinforcing the belief that such foods were harmful or incompatible with their bodies. Daniel described strong physical reactions after eating fast food:

*“Anytime I do have any of that… KFC or kebab… you feel it. I feel that I’ve had that… digestion-wise I feel it on my chest… like something is just sitting there.”*

Here, bodily sensations become interpreted as confirmation that certain foods are incompatible with the body. Such interpretations reinforce dietary rules by framing deviation as something the body itself rejects. Similarly, participants who had eliminated particular food groups described the idea of reintroducing them as both physically and morally unsettling. Oliver explained how abstaining from meat over time transformed their perception of it:

*“Now because I haven’t eaten it for so long the idea of eating meat is really alien to me… I would feel wrong.”*

This sense of “wrongness” suggests that dietary practices had become internalised as part of participants’ moral and embodied identities. Food choices therefore extended beyond nutrition, shaping how participants understood themselves and their relationship to their bodies. When participants did deviate from their dietary rules, these moments often triggered feelings of guilt and a perceived need for compensatory behaviours. Aisha described how eating confectionary would lead to efforts to restore balance through increased physical activity:

*“Sometimes it happens, I have one… but then I feel utterly guilty… so the next week I would probably walk to work four times a week.”*

In this account, exercise functions as a mechanism of moral repair, allowing participants to reassert control and restore a sense of equilibrium. Similarly, Anna described more restrictive responses following dietary lapses:

*“If I eat something outside of my rules, I have to cut out meals basically… starve some days just to give myself a lesson.”*

These narratives highlight how food and exercise practices can become intertwined within a system of self-regulation in which deviation prompts corrective behaviours aimed at restoring moral order. Beyond dietary transgressions, disruptions to exercise routines also appeared to trigger emotional responses. Sofia described how missing exercise affected their mood and self-perception:

*“If I miss a lot of exercise… I get really grumpy… you start feeling bad about yourself.”*

In this way, bodily practices such as diet and exercise appear to function as key mechanisms through which participants maintain a sense of control, self-worth, and identity. At the same time, participants’ accounts also revealed a contrasting pattern, in which rigid moral frameworks around food were gradually softened or renegotiated. Rather than positioning all deviations as failures requiring correction, some participants described moving toward a more flexible and self-compassionate approach to eating. Hannah reflected on shifting away from earlier patterns of self-criticism:

*“I used to beat myself up for diverging but now I try not to be so strict… I say I’m human, there will be moments like that.”*

Similarly, James emphasised the importance of intentionality and enjoyment in eating decisions:

*“If I make a decision I stick to it… you have to feel happy while you eat, feel grateful.”*

These accounts suggest processes of moral repositioning in which previously rigid dietary rules are reinterpreted and integrated into a more flexible framework. While less dominant within the dataset, such narratives indicate that participants are not uniformly constrained by moralised food rules, but may actively negotiate and, in some cases, resist them. This pattern points to a potential trajectory of change that warrants further exploration, particularly in relation to how individuals transition from rigid to more adaptive relationships with food over time.

## 4. Discussion

The present study sought to explore the moral and self-worth meanings underlying ON-related eating practices through qualitative analysis. Four interrelated themes were identified, suggesting that ON-related behaviours are not solely about food choices but are embedded within broader processes of identity construction, moral meaning-making, and self-regulation. The findings further support the view that ON may reflect complex sociocultural dynamics surrounding health, morality, and self-optimisation rather than a distinct psychiatric condition.

### 4.1. Discipline, Self-Control, and the Moralization of Health

Participants’ narratives consistently framed healthy eating as a matter of willpower, mental strength, and personal control. Maintaining a strict diet was not described merely as a practical health behaviour but as evidence of psychological discipline and self-mastery. Participants frequently positioned themselves as individuals capable of “pushing themselves,” training their minds, and regulating bodily desires in ways that signified strength of character. In this sense, dietary restraint was constructed not simply as a nutritional practice but as a moral and psychological achievement.

These narratives resonate with previous qualitative research suggesting that individuals with orthorexic tendencies often interpret restrictive dietary practices as expressions of self-discipline and responsibility rather than as forms of disordered eating (Fixsen et al., 2020; McGovern et al., 2020). Rather than viewing their behaviours as excessive, participants frequently framed them as desirable forms of self-improvement that extend beyond food practices into other areas of life. The capacity to control one’s diet was therefore presented as evidence of broader personal competence, reinforcing the idea that disciplined eating reflects a disciplined self (Tragantzopoulou et al., 2026a; Tragantzopoulou et al., 2026b).

From a sociocultural perspective, such accounts mirror wider neoliberal discourses that position health as an individual responsibility and a marker of moral worth. Within the ideology of healthism, individuals are expected to actively manage their bodies through lifestyle choices, transforming health into a personal project of self-regulation and optimisation (Crawford, 1980; Kristensen et al., 2016). Participants’ narratives closely reflected this logic: dietary restraint was framed not simply as a strategy for wellbeing but as a demonstration of determination, commitment, and personal strength. In this context, the ability to adhere to strict food rules becomes a symbol of moral virtue, while failure to do so may be interpreted as a lack of discipline or moral inferiority.

These dynamics can also be interpreted through a Foucauldian lens of disciplinary power and technologies of the self. Foucault argued that modern forms of power operate not primarily through external coercion but through the internalisation of norms that lead individuals to regulate their own behaviour (Foucault, 1977, 1988). Participants’ descriptions of monitoring their diets, training their minds, and pushing their bodies to maintain strict eating practices illustrate how such processes of self-surveillance may operate in relation to food. Rather than being imposed externally, dietary discipline appears to be actively embraced as part of a broader project of self-formation.

Importantly, this framing may help explain why restrictive dietary behaviours were rarely described by participants as problematic. Instead, they were often interpreted as evidence of commitment to self-improvement and personal growth. In contemporary wellness cultures, practices such as clean eating, fasting, and strict nutritional regulation are frequently celebrated as signs of dedication and self-control, despite being linked to disordered eating (Ambwani et al., 2019; Tragantzopoulou et al., 2024a). As a result, behaviours that might otherwise be considered rigid or restrictive can be reframed as admirable demonstrations of discipline. This reinforces the difficulty of distinguishing between socially endorsed health practices and behaviours that may become psychologically burdensome or restrictive.

This framing also echoes broader psychological accounts of extreme dietary regulation, where rigid self-control over food is conceptualised as part of a continuum of ascetic or self-denying behaviours historically associated with moral purification and self-transcendence (Rampling, 1985; Vitousek et al., 1998). Within this continuum, in some of our participants’ accounts, dietary restraint may be interpreted as taking on symbolic meanings that extend beyond health into domains of virtue, purity, and moral elevation.

### 4.2. Purity, Health, and the Body as Moral Proof

Participants frequently positioned the body as visible confirmation that their dietary practices were correct. Improvements in skin appearance, digestion, energy levels, and general bodily functioning were described as evidence that their food choices were working. In this way, the body operated as a form of proof—an external marker through which internal discipline, purity, and health were made visible.

Such interpretations align with previous research indicating that individuals engaged in restrictive healthy eating often interpret bodily changes as validation of dietary success (Barthels et al., 2018). However, the present findings suggest that these bodily outcomes functioned not merely as indicators of physical health but as moral confirmation. Participants described their bodies as evidence that their lifestyle was superior to that of others who were perceived as less disciplined or less attentive to their health. Within these narratives, bodily appearance and functioning became symbolic markers of virtue, self-control, and personal responsibility.

This dynamic reflects what Rozin (1999) describes as the moralization of food, whereby dietary choices become infused with moral meaning. When food practices are moralised, eating “correctly” becomes associated with virtue, while consuming certain foods may be interpreted as a sign of personal failure, weakness, or lack of self-control. Participants’ accounts in the present study illustrated this process clearly, particularly in descriptions of individuals who consume “unhealthy” foods as lacking discipline or producing unpleasant bodily effects such as body odour or digestive discomfort. These narratives highlight how dietary practices can become a basis for moral judgement and social differentiation.

Importantly, such moral hierarchies around food are closely tied to broader cultural valuations of bodies. Within contemporary health discourses, lean, controlled, and visibly “fit” bodies are frequently positioned as evidence of discipline and moral responsibility, while larger bodies are often stigmatised as indicators of laziness, lack of self-control, or moral failure (Kwan & Graves, 2013; Peng, 2025; Tragantzopoulou & Giannouli, 2025). Research on weight stigma demonstrates that individuals in larger bodies frequently experience discrimination and social judgement, including in workplaces, healthcare settings, and everyday social interactions (Bannuru, 2025; Tragantzopoulou, 2025). Within this cultural context, the pursuit of dietary purity may also function as a strategy for distancing oneself from the stigma associated with bodies perceived as unhealthy or undisciplined.

From this perspective, orthorexic practices can be understood not only as attempts to achieve physical health but also as efforts to produce a socially valued body that communicates discipline, control, and moral worth. The body becomes a site through which individuals demonstrate adherence to dominant health norms and signal their commitment to responsible self-care (Kickbusch, 1986; Kristensen et al., 2016). Such dynamics reinforce the idea that ON operates within broader cultural frameworks that reward visible bodily discipline while simultaneously stigmatising bodies that deviate from these ideals.

A further dimension that warrants consideration is the role of gender in shaping these narratives of purity, discipline, and bodily control. Although the present study was not explicitly designed to examine gender differences, the predominance of women in the sample (13 out of 18 participants), alongside the presence of participants working in appearance-focused professions such as modelling, suggests that these constructions may be situated within gendered cultural expectations. Existing literature indicates that healthism and body discipline are often more intensely imposed on women, who are subject to heightened scrutiny regarding appearance, self-control, and bodily regulation (Kwan & Graves, 2013; Tragantzopoulou, 2025). Within this context, the emphasis on dietary purity and bodily “proof” observed in participants’ accounts may reflect not only individual health beliefs but also broader gendered pressures to embody ideals of discipline, cleanliness, and aesthetic control.

Finally, these findings resonate with Fischler’s (1988) argument that food plays a central role in both individual and collective identity formation. By selectively incorporating certain foods and rejecting others, individuals symbolically construct both their bodies and their identities. In the present study, dietary practices functioned as a form of embodied identity work, through which participants not only attempted to optimise their health but also produced a body that reflected their values, moral commitments, and sense of self.

### 4.3. Ethical Consumption and Moral Identity

A third key finding concerns the role of ethical discourses in shaping participants’ dietary practices, particularly among those following vegan or predominantly plant-based diets. Participants frequently described their eating behaviours as motivated not only by concerns about health but also by ethical commitments related to animal welfare, environmental sustainability, and social responsibility. In these accounts, dietary choices were framed as expressions of broader moral values rather than merely personal health strategies. These findings are consistent with previous research indicating that vegan and vegetarian diets are often motivated by multiple intersecting concerns, including ethical, environmental, and health-related considerations (Barthels et al., 2018; Szulc et al., 2025). Some studies have suggested that ethical motivations may function as protective factors against pathological eating behaviours (Barthels et al., 2018), as they shift the focus away from body control and towards political or moral commitments beyond the self. However, the present findings complicate this interpretation by suggesting that ethical motivations may also contribute to the moral intensification of dietary practices. Participants frequently framed their food choices as morally preferable to mainstream eating habits, positioning their dietary practices as more responsible, compassionate, or environmentally conscious. In this sense, dietary restriction was not experienced simply as self-denial but as an expression of ethical integrity. Such narratives appeared to reinforce adherence to highly controlled eating practices by embedding them within a broader moral worldview in which food choices become symbolic demonstrations of virtue and responsibility.

From this perspective, participants’ accounts suggest that orthorexic tendencies may be experienced as shaped not only by concerns about health but also by the moral significance attached to dietary decisions. When food practices become linked to ethical identity, deviations from these practices may be experienced not merely as dietary lapses but as moral failures (Lakritz et al., 2022; Horovitz, 2025). This dynamic may help explain the rigidity sometimes observed in orthorexic eating patterns, as dietary rules become intertwined with individuals’ sense of moral selfhood. More broadly, these findings reflect contemporary cultural trends in which food increasingly functions as a marker of identity, values, and social belonging. As Fischler (1988) argues, food practices often operate as symbolic boundaries through which individuals distinguish themselves from others. Within this framework, veganism and other restrictive dietary practices may serve not only nutritional or ethical purposes but also identity-affirming functions. For individuals displaying orthorexic tendencies, such practices may therefore operate simultaneously as health behaviours, ethical commitments, and mechanisms through which moral identity is constructed and maintained.

These dynamics may also be understood in relation to broader historical and cultural traditions in which dietary restriction has been associated with moral purity, asceticism, and spiritual discipline. Historical analyses of AN highlight how extreme forms of food restriction have long been embedded within ascetic and religious traditions, where fasting and bodily denial were linked to spiritual elevation and moral virtue, as seen in accounts of mediaeval “holy anorexia” (Behar & Arancibia, 2015). Such perspectives suggest that contemporary restrictive eating practices may echo older cultural logics in which control over the body is imbued with moral or quasi-spiritual significance, even if the underlying aims differ.

In a similar vein, contemporary research on EDs has identified asceticism as a relevant psychological and personality-related construct associated with dietary restraint, perfectionism, and overcontrol (Obeid et al., 2021). Traits such as self-discipline, self-sacrifice, and rigid goal-directed control have been linked to more severe ED presentations, highlighting how moralised self-regulation may operate across both clinical and subclinical eating-related behaviours.

Within the present findings, these broader tendencies toward moralised control may also intersect with culturally or religiously informed dietary frameworks. For example, participants referencing structured dietary rules (such as halal practices) may reflect how pre-existing moral or religious food regulations can coexist with, and potentially reinforce, disciplined eating orientations, without being inherently pathological. This suggests that strict dietary practices may acquire meaning through multiple overlapping moral systems, rather than a single psychological explanation. As such, participants’ accounts suggest that orthorexic tendencies may be experienced as shaped not only by concerns about health but also by the moral significance attached to dietary decisions.

### 4.4. Moral Failure, Guilt, and the Repair of the Self

The final theme, “guilt and moral repositioning,” highlights the emotional consequences associated with deviating from dietary rules. Participants described experiencing guilt when they failed to adhere to their dietary standards, often attempting to compensate through increased exercise or further dietary restriction. These findings are consistent with previous qualitative research indicating that orthorexic behaviours are frequently accompanied by feelings of guilt and anxiety when dietary rules are violated (Cheshire et al., 2020; Zickgraf, 2020). However, the present findings suggest that guilt may operate not only as an emotional response to dietary deviation but also as part of a broader moral framework governing food consumption. In this context, dietary transgressions appear to trigger a process of moral repositioning, whereby individuals attempt to restore their sense of moral integrity through compensatory behaviours such as increased exercise or stricter dietary control. Such responses indicate that dietary rules are not experienced merely as health guidelines but as moral obligations. When these obligations are violated, individuals engage in corrective practices aimed at re-establishing their identity as disciplined and responsible subjects.

This dynamic reflects the internalisation of moralised health norms, whereby individuals continuously monitor and evaluate their behaviour according to perceived standards of “good” or “correct” eating. Guilt appeared to function as a mechanism of self-regulation that reinforced adherence to restrictive dietary practices. Rather than disrupting orthorexic behaviours, participants’ accounts suggest that feelings of guilt may therefore contribute to their maintenance by motivating further efforts to regain moral and bodily control. The role of guilt in maintaining restrictive eating behaviours has been observed in other eating-related conditions, particularly in relation to rigid food rules and compensatory behaviours (Raffone et al., 2025). However, in the context of ON, guilt appears to be linked not only to concerns about body image or weight but also to broader moral ideals surrounding purity, health, and self-discipline. This suggests that orthorexic eating patterns may be sustained not only through concerns about physical health but also through the moral meanings attached to food and the body. This interpretation is consistent with broader literature on extreme self-regulation and eating pathology, which conceptualises rigid dietary control and associated guilt responses as part of a continuum of ascetic and compulsive behaviours characterised by moralised self-denial and heightened self-surveillance (Rampling, 1985; Vitousek et al., 1998).

Importantly, these findings can also be interpreted in light of emerging cognitive-psychopathological models of ON. Quantitative research has demonstrated that the relationship between dieting behaviours, obsessive-compulsive traits, and orthorexic symptomatology is partially mediated by persistent food-related preoccupations and worries (Rossi et al., 2024). From this perspective, the pattern described by participants in the present study—whereby dietary deviation triggers guilt followed by corrective behaviours—may be interpreted as reflecting not only moral self-regulation but also underlying cognitive processes characterised by intrusive thoughts, heightened monitoring, and preoccupation with food. Similarly, network analytic evidence suggests that emotional consequences related to eating, worry about healthy food, and obsessive thinking act as “bridge symptoms” linking ON with both ED and obsessive-compulsive symptom clusters (Lombardo et al., 2026).

Integrating these perspectives, the present findings suggest that guilt may operate at the intersection of sociocultural and cognitive mechanisms. While participants’ accounts highlight the moralization of food and the construction of dietary discipline as a marker of self-worth, these processes may be reinforced and maintained through cognitive patterns of preoccupation and rumination. In this sense, the “moral repair” processes described by participants may also be understood as a means to alleviate distress associated with intrusive food-related thoughts or perceived loss of control. This integrative interpretation extends existing literature by demonstrating how culturally embedded moral meanings around health and purity may interact with transdiagnostic cognitive mechanisms, contributing to the persistence of restrictive eating practices. This interpretation is further supported by qualitative research with clinicians, which suggests that dietary behaviours in eating-related conditions are often embedded within broader transdiagnostic processes, including perfectionism, control, and emotion regulation difficulties, and are experienced as emotionally and symbolically meaningful rather than purely nutritional practices (Tragantzopoulou et al., 2026b). As such, the compensatory and “repair” behaviours described by participants may also reflect attempts to manage emotional discomfort and restore a sense of psychological equilibrium, rather than solely adherence to moralised health norms.

### 4.5. Implications and Novel Contributions

The findings of this study contribute to a growing body of literature suggesting that ON cannot be fully understood as a purely individual pathology. Rather, orthorexic practices appear to be embedded within broader sociocultural frameworks that emphasise health optimisation, moral responsibility, and identity construction through food (Fixsen et al., 2025b). Participants’ narratives illustrate how dietary practices become intertwined with projects of self-construction, whereby individuals seek to cultivate discipline, demonstrate moral integrity, and express ethical values through their eating behaviours. Within this context, restrictive dietary practices are rarely interpreted by individuals themselves as problematic. Instead, they are framed as evidence of psychological strength, bodily purity, and ethical commitment. These findings highlight how orthorexic tendencies may be reinforced by contemporary cultural discourses that celebrate self-control, bodily optimisation, and “clean” lifestyles. As a result, behaviours that might otherwise be considered restrictive or excessive may become socially validated, complicating efforts to distinguish between culturally endorsed health practices and potentially harmful patterns of eating.

The present study extends previous research in several important ways. First, it highlights how discipline and mental control function as central elements in participants’ narratives, suggesting that orthorexic practices may operate as forms of self-regulation and identity management rather than solely as expressions of dietary concern. Second, the findings illustrate how the body becomes a form of moral evidence, through which individuals interpret physical changes as validation of their dietary practices and personal virtue. Third, the study demonstrates how ethical motivations, particularly in relation to vegan or plant-based eating, may intersect with processes of moral identity construction, reinforcing commitment to restrictive diets. Finally, the analysis shows how feelings of guilt following dietary transgressions contribute to processes of moral repositioning that help sustain dietary control. Together, these findings provide further evidence that ON-related behaviours are deeply intertwined with systems of moral meaning, identity work, and cultural expectations surrounding health and the body. Understanding orthorexia therefore requires moving beyond purely clinical frameworks to consider the broader sociocultural contexts in which such practices are produced and maintained.

### 4.6. Limitations and Strengths

Some limitations should be acknowledged. As with most qualitative research, the relatively small sample size limits the extent to which findings can be generalised to wider populations. Participants were individuals who self-identified as engaging in strict healthy eating practices, and their narratives may therefore reflect particular personal experiences and sociocultural perspectives. Additionally, the study was conducted within the UK, meaning that cultural factors specific to this context may have shaped participants’ understandings of health, diet, and morality. A further limitation concerns the conceptual framing of the sample. Although participants were recruited based on self-identification as individuals strongly adhering to healthy eating practices, and an informal set of screening criteria was applied during initial contact to ensure consistency, no validated screening tool for ON was administered. Given that ON is not a formally recognised diagnosis and is widely understood to exist on a continuum with socially accepted “healthy” eating behaviours, the findings should not be interpreted as representing clinically defined orthorexia. Instead, the sample reflects individuals who engage in restrictive or highly regulated healthy eating practices. Therefore, references to orthorexia-related tendencies should be understood as interpretative and theoretically informed, rather than diagnostic, and as situated within broader sociocultural constructions of health and morality. Finally, the study included a higher proportion of women, and although this reflects trends in research on health-focused eating practices, it may have shaped the prominence of themes related to discipline, bodily control, and appearance. The relatively small number of male participants limits the extent to which gendered differences could be explored in depth. Future research would benefit from more gender-balanced or explicitly gender-focused samples in order to examine how orthorexic tendencies and the moralization of food may be differently constructed across genders.

At the same time, the study also presents several strengths. The sample included eighteen participants ranging in age from 19 to 58 years and included both women and men from diverse backgrounds, allowing for a broad range of perspectives on healthy eating practices. The use of in-depth semi-structured interviews provided rich accounts of participants’ experiences and allowed for a detailed exploration of the meanings individuals attribute to their dietary practices. Furthermore, the qualitative design enabled an in-depth examination of the moral and identity-related dimensions of orthorexic behaviours, offering insights that may not be easily captured through quantitative approaches. Future research could further explore how orthorexic behaviours are shaped across different cultural contexts and social environments, particularly in relation to social media influences, wellness cultures, and fitness communities. Longitudinal qualitative studies may also provide valuable insight into how individuals’ relationships with food and health evolve over time.

## 5. Conclusions

The present study highlights how ON-related practices are embedded within broader sociocultural narratives that frame health, discipline, and bodily control as markers of moral worth. Participants’ accounts reflect that restrictive dietary practices may function not only as attempts to achieve physical health but also as forms of identity construction and moral self-regulation. Within contemporary wellness cultures that emphasise personal responsibility for health, such practices can become socially validated and even admired, potentially obscuring the boundary between health-conscious behaviour and disordered eating.

These findings have important implications for how ON is conceptualised and addressed in both research and clinical settings. If restrictive dietary practices are embedded within moral beliefs about purity, discipline, and ethical responsibility, individuals may be less likely to perceive their behaviours as problematic or in need of intervention. This may add further complexity to treatment, particularly given that EDs are already recognised as challenging conditions to treat (Tragantzopoulou & Giannouli, 2023; Tragantzopoulou et al., 2024b). Addressing orthorexic tendencies may therefore require approaches that not only focus on eating behaviours themselves but also engage with the underlying moral frameworks and identity processes through which individuals make sense of their dietary practices.

Overall, understanding ON requires moving beyond a narrow focus on individual pathology to consider the broader cultural, moral, and social contexts that shape contemporary relationships with food, health, and the body.

## Figures and Tables

**Table 1:** Demographic characteristics and self-reported dietary preferences of participants (N = 18).

Participant ID *	Sex	Age (Years)	Ethnicity	Self-Reported Dietary Preferences	Employment
Daniel	Male	32	Black African	Halal	Self-employed
Oliver	Male	24	White British	None	Student/Carer
Sofia	Female	25	Other White	Vegan	Psychology researcher
Aisha	Female	35	Other Asian	Vegan	Paralegal
Maya	Female	29	Other Asian	Vegetarian	Model
Chloe	Female	24	Other White	Predominantly plant-based with occasional chicken	Model
Laura	Female	23	White British	Ovo-vegetarian/flexitarian	Model
Victoria	Female	33	White British	None	Make-up artist
Isabella	Female	27	Mixed	None	Retail (high-end fashion brand)
Natalie	Female	31	Other White	None	Model
Ethan	Male	30	White British	Vegetarian	Personal trainer
Priya	Female	28	British Asian	Vegan	Marketing executive
James	Male	58	White British	None	IT consultant
Anna	Female	26	Black British	None	Graduate student
Hannah	Female	37	White British	Vegetarian	Yoga instructor
Lucas	Male	29	Mixed	Vegan	Photographer
Amira	Female	32	Middle Eastern	None	Nurse
Zoe	Female	22	White British	Vegan	University student

**Table 2:** Overview of themes identified in the reflexive thematic analysis.

Theme	Description	Illustrative Focus
**1. The Disciplined Self**	Participants constructed healthy eating as a product of mental strength, discipline, and self-mastery. Dietary restraint and fasting were framed as tests of willpower that extended beyond food and reflected broader self-control in life.	Mental strength, self-control, training the mind, discipline in eating and exercise
**2. The Body as Evidence of Purity and Health**	Participants interpreted bodily changes as evidence that their dietary practices were beneficial. Physical markers such as skin quality, digestion, energy levels, weight changes, and bodily odour were understood as indicators of internal purity or imbalance.	Skin improvement, digestion, weight loss, energy, bodily cleanliness, smell
**3. Ethical Eating and Moral Positioning**	Food choices were embedded within moral and ethical frameworks, particularly around animal welfare and environmental or social responsibility. Participants negotiated these ethical commitments while also acknowledging social tensions associated with moralised eating practices.	Veganism, vegetarianism, animal rights, ethical awareness, social judgement
**4. Guilt and Moral Repositioning**	Deviations from dietary rules triggered guilt, bodily discomfort, and compensatory behaviours such as increased exercise or dietary restriction. However, some participants also described attempts to adopt a more flexible stance toward food and self-discipline.	Guilt after “unhealthy” food, compensatory exercise, restriction, self-forgiveness

## Data Availability

Data can be made available upon reasonable request.

## Abbreviations

The following abbreviations are used in this manuscript:

ON	Orthorexia Nervosa
EDs	Eating Disorders
UK	United Kingdom

## Appendix A

The COREQ Checklist

**Domain**	**Item No.**	**Guide Questions/Description**	**Reported on Page No.**
**Domain 1: Research team and reflexivity**			
Personal characteristics			
Interviewer/facilitator	1	Which author(s) conducted the interview or focus group?	8-10
Credentials	2	What were the researcher’s credentials (e.g., PhD, MD)?	8-10
Occupation	3	What was their occupation at the time of the study?	8-10
Gender	4	Was the researcher male or female?	8-10
Experience and training	5	What experience or training did the researcher have?	8-10
Relationship with participants			
Relationship established	6	Was a relationship established prior to study commencement?	8
Participant knowledge of interviewer	7	What did participants know about the researcher (e.g., goals, motivations)?	8-9
Interviewer characteristics	8	What characteristics were reported about the interviewer (e.g., assumptions, biases, interests)?	8-9
**Domain 2: Study design**			
Theoretical framework			
Methodological orientation and theory	9	What methodological orientation underpinned the study (e.g., phenomenology, grounded theory)?	6
Participant selection			
Sampling	10	How were participants selected (e.g., purposive, convenience, snowball)?	6-7
Method of approach	11	How were participants approached (e.g., face-to-face, email)?	6-7
Sample size	12	How many participants were included in the study?	6-7
Non-participation	13	How many declined or dropped out, and why?	6-7
Setting			
Setting of data collection	14	Where was the data collected (e.g., home, online, workplace)?	8-9
Presence of non-participants	15	Were others present during data collection?	8-9
Description of sample	16	What are key characteristics of the sample (e.g., demographics)?	7-8
Data collection			
Interview guide	17	Were interview questions or guides provided or pilot tested?	8-9
Repeat interviews	18	Were repeat interviews conducted? If yes, how many?	8-9
Audio/visual recording	19	Was audio or video recording used?	8-9
Field notes	20	Were field notes taken during or after interviews?	8-9
Duration	21	What was the duration of interviews or focus groups?	8-9
Data saturation	22	Was data saturation discussed?	6
Transcripts returned	23	Were transcripts returned to participants for feedback or correction?	8-9
**Domain 3: Analysis and findings**			
Data analysis			
Number of data coders	24	How many researchers coded the data?	9-10
Description of coding tree	25	Was a coding tree described?	9-10
Derivation of themes	26	Were themes pre-defined or derived from data?	9-10
Software	27	What software was used for data analysis?	9-10
Participant checking	28	Did participants provide feedback on findings?	9-10
Reporting			
Quotations presented	29	Were participant quotations used and identified?	11
Data and findings consistency	30	Was there consistency between data and findings?	11
Clarity of major themes	31	Were major themes clearly presented?	11
Clarity of minor themes	32	Were minor themes or variations discussed?	11
